# Ocular disorders among persons living with HIV/AIDS in Osun State, Nigeria: a cross-sectional study

**DOI:** 10.1186/s12886-026-04689-w

**Published:** 2026-03-11

**Authors:** Olubayo U. Kolawole, Olufunmilayo I. Fawole, Oyindamola B. Yusuf, Olawale. J. Oladejo

**Affiliations:** 1 Department of Surgery, College of Health Sciences, Bowen University, Iwo, Nigeria; 2 Department of Epidemiology and Medical Statistics, University of Ibadan, Ibadan, Nigeria; 3 Department of Surgery, University of Medical Sciences, Ondo, Nigeria; 4 Department of Ophthalmology & Visual Sciences, University of British Columbia, Vancouver, BC Canada

**Keywords:** Human immunodeficiency virus, Acquired immune deficiency syndrome, Ocular disorders, Cytomegalovirus retinitis, Herpes zoster ophthalmicus, Osun, Nigeria

## Abstract

**Background:**

HIV/AIDS affects all the structures of the eye and the orbit with grave implications on vision and quality of life. More than half of persons living with HIV/AIDS (PLWHA) are at risk of developing at least one HIV-related ocular disorder in a lifetime. This study aims to determine the prevalence, spectrum, and correlates of ocular disorder among PLWHA in Osun State, Nigeria.

**Methods:**

A multicentre cross-sectional study of a randomly selected sample of PLWHA in Osun State. Participants were interviewed using semi-structured questionnaires and underwent detailed ophthalmologic clinical examinations. The outcome variables were presence or absence of ocular disorders and visual impairment. Descriptive statistics, Chi square test and binary logistic regression were used to analyse data using SPSS version 20.

**Results:**

A total of 505 PLWHA (80% female) aged 3.5–80 years (median, 40.0) were enrolled. They had lived with HIV/AIDS for a median period of 3.0 years (range: 0-12.8), and over 90% were on highly active anti-retroviral therapy. Their median CD4 + T lymphocyte count was 400 cells/ µl (range: 8-1977). Prevalence of any ocular disorder was 53.9% (95% C.I. =49.6%-58.2%), while the prevalence of HIV-related ocular disorders was 5.4% (95% C.I. =3.4%-7.4%). Common HIV-related ocular disorders were uveitis (1%), presumed toxoplasmosis (0.8%) and neuro-ophthalmic disorders (0.8%), and Cytomegalovirus (CMV) retinitis (0.4%). Prevalence of visual impairment in the sample was 4.4%, and refractive errors (50.0%) and cataract (13.6%) were the common causes. Participants with CD4 + count of at least 500 cells/µl (OR = 0.24; 95% C.I.=0.07–0.89; *p* = 0.03); those without visual impairment (OR = 0.25; 95% C.I.=0.08–0.80; *p* = 0.02) and those without eye complaints (OR = 0.35; 95% C.I.=0.15–0.83; *p* = 0.02) were less likely to have HIV-related ocular disorders.

**Conclusions:**

Ocular disorders were common among PLWHA in Osun State although the prevalence of HIV- related ocular disorders was low. Moreover, there appeared to be emergence of previously rare and potentially blinding disorders such as CMV retinitis.

**Supplementary Information:**

The online version contains supplementary material available at 10.1186/s12886-026-04689-w.

## Introduction

The scourge of Human immunodeficiency Virus/Acquired immunodeficiency syndrome (HIV/AIDS) has had a negative impact on physical, psychological and socio-economic well-being of world populations since its pandemic began in 1981 [1]. HIV/AIDS is a multi-systemic disease, affecting all parts of the human body including the eye and the visual system. Ocular disorders in HIV/AIDS were first reported in the US in 1982 by Holland and co-workers [2]. It was suggested that HIV-related ocular disorders could affect about 50–75% of persons living with HIV/AIDS (PLWHA) at some points in the course of their illnesses [3]. Ocular disorders in PLWHA result from the direct effects of the virus on ocular structures and immunosuppression (through development of opportunistic infections and certain cancers). In addition, the eyes are involved in immune reconstitution inflammatory syndrome (IRIS), which develops in patients who commence highly active anti-retroviral therapy (HAART) when they are very immune-deficient. IRIS results in clinical deterioration of pre-existing opportunistic infections owing to rapid restoration of dysregulated immune response against pathogen-specific antigens [4].

Geographic and temporal variations in the spectrum of ocular complications of HIV/AIDS have been documented. In the pre- HAART, CMV retinitis was commoner in the developed countries and South Asia than the sub-Saharan Africa [5–7]. On the other hand, herpes zoster ophthalmicus (HZO) was commoner in the sub-Saharan Africa than the developed countries such that it was postulated to be a marker for HIV infection in the younger age groups with a high predictive value [8]. The observed differences had been attributed to earlier and higher mortality among PLWHA in developing countries than developed ones; racial differences; differences in the HIV subtypes that infected PLWHA in both regions and co-morbid diseases [9]. During the HAART era, there have been significant shift in ocular morbidity associated with HIV/AIDS worldwide. For instance, Nigeria, other parts of Africa, and Asia have witnessed significant reductions in occurrence of HZO [10–15]. Similarly, the developed countries like the United States have had significant reduction in CMV retinitis [16, 17]. However, the advent of HAART caused a surge in the prevalence of retinal microangiopathy, HIV retinopathy, conjunctival microvasculopathy, dry eye syndrome, and uveitis. Ironically, CMV retinitis now appear more among African patients.

HIV/AIDS related ocular disorders could result in visual impairment. It has been estimated that between 8.6% and 23.3% of Nigerians living with HIV/AIDS are expected to be visually impaired [18, 19]. Studies have also shown that low CD4 + T-lymphocyte counts [20], high viral load [12], advanced stage of HIV infection [21], self-reported eye problems and low visual acuity [22] were important predictors of HIV related ocular disorders.

The greatest burden of HIV in Sub-Saharan Africa is borne by Nigeria, where about 2 million out of approximately 40.8 million PLWHA globally lived in 2024 [23]. There were about 60,000 PLWHA in Osun State, Nigeria who could be at risk of developing visual impairment and other ocular complications of HIV/AIDS [24]. The magnitude, spectrum and visual consequences of ocular disorders among PLWHA in Nigeria has not been comprehensively described, especially in the HAART era. However, the few available reports in Nigeria showed that prevalence of HIV related ocular disorders ranged between 7.1% in Benin, South-South, Nigeria [10] and 14.3% in Owo, South West, Nigeria [25]. Data are sparse concerning the burden of ocular diseases among PLWHA and their consequences in Osun State, Nigeria. Therefore, this study determined the prevalence, spectrum of ocular disorders, and their correlates among PLWHA in Osun State, Nigeria.

## Subjects and methods

### Study settings and patients

This multi-centre cross-sectional study was carried out at seven out of the 14 Anti-retroviral treatment (ART) centres in Osun State, Nigeria between November 2015 and February 2016. Participants who had been confirmed HIV positive and were receiving care at those ART centres during the study period formed the study population. Patients who had tested negative for HIV or indeterminate were excluded from the study.

### Sample size determination

The estimated sample size for this study was 450, assuming prevalence of ocular disorders among PLWHA = 0.785 (from a previous study in Lagos, Nigeria [18]), Z_α/2_ = 1.96, degree of accuracy = 4% and non-response rate = 10%.

### Sampling technique

All the 14 ART centres in Osun State were stratified into 3 groups based on the senatorial district in which they were located. At least one ART centre was selected from each senatorial district by simple random sampling technique using computer generated random numbers. All consenting PLWHA irrespective of HAART treatment and ocular statuses were enrolled from Ede, Ejigbo, Ife (2 ART centres), Ilesha, Iwo, and Osogbo ART centres until the sample size was attained.

### Data collection procedures

Socio-demographic data such as age, gender, occupation, marital status, ethnicity, educational level, family structure were collected. Other information such as knowledge of HIV status, HAART treatment status, past and present eye symptoms were also collected. In addition, WHO clinical stage of HIV infection, HAART regimen and other drugs being received, co-morbid systemic and/or sexually transmitted diseases, and recent CD4 + count were extracted from respondent's care cards.

Thorough ophthalmic examinations were conducted by an ophthalmologist (OUK). Visual acuity (VA) was assessed with Snellen's chart in each eye of each participant at six or three metres by a trained research assistant (RA), who was recruited from the research unit of the Osun State University Teaching Hospital (UNIOSUNTH). In each of the ART centre, OUK re-checked VA of one in every 20 respondents to ensure good agreement. Visual acuity was assessed unaided or with glasses (if subject was using one). All eyes with VA less than 6/18 had their VA re-assessed with pinhole. Subjects who could not see size 60 alphabet of the Snellen's chart at 3 m were further assessed to determine whether they could perceive light in the affected eyes. If they did, their VA was adjudged to be “perception of light (PL); the VA of respondent who did not perceive light was considered “no perception of light” in the affected eye(s). In each ART centre, the ophthalmologist re-checked VA of one in at least every 20 respondents to ensure good agreement. There was perfect agreement between the RA and OUK.

The ocular adnexal structures and the anterior segment of each eye, which included the lids, lashes, conjunctiva, cornea, anterior chamber, pupil and the lens were examined by the ophthalmologist using the bright light from binocular indirect ophthalmoscope and the magnification of + 22 D Volk lens. Slit lamp examination of the anterior segments was done only when indicated. In addition, ocular alignment and motility were assessed in each eye. Intraocular pressures were measured with Perkins Applanation tonometer when indicated. Posterior segments of each participant's eyes were assessed after dilating the pupils with a combination of tropicamide 1% and phenylephrine 5% eye drops and waiting for 10–20 min. The ocular fundi were thereafter examined with binocular indirect ophthalmoscope and + 22D Volk lens. Slit lamp biomicroscopy with + 78D Volk lens was done when indicated. All examination instruments were handled with care by the ophthalmologist, and no harm was inflicted on any participant. All data collected were recorded in the interviewer administered semi-structured questionnaire, which was specifically designed for this study (Supplementary file). All participants who required further evaluation, treatment or follow up were referred to the eye clinic of Osun State University Teaching Hospital, Osogbo.

### Ethical considerations

This study was conducted according to the principles expressed in the Declaration of Helsinki. The study was approved by the Ethical and Research Committee of UNIOSUNTH, Osogbo (LTH/EC/2016/02/261). Informed consent was obtained from each participant after providing information about the study.

### Data analysis

Data entry and analysis were done using SPSS version 20 software (SPSS, Inc., IL, USA). Chi-squared tests and Fisher's exact tests (where appropriate) were used to compare proportions. Binary logistic regression was performed to examine associations between ocular disorders in HIV/AIDS and respondents' socio-demographic and clinical characteristics. Similarly, the associations between visual impairments and patients' socio-demographic and clinical characteristics were examined. All analyses were carried out at 5% level of significance.

## Results

### Socio-demographic and clinical characteristics of respondents

Study participants comprised 101 males and 404 females, giving male: female ratio of 1:4 (Table 1). The median age of participants was 40.0 years (range: 3.5–80). Most (88.7%) of the participants were urban residents. Majority (87.3%) were married. Further, most (93.1%) of the participants belonged to the Yoruba ethnic nationality. Most (85.5%) of the participants had WHO clinical stages 1 and 2 HIV disease (Table 2). HIV 1 was the commonest HIV strain that infected the participants, accounting for 99.6%. More than three-quarters (76.4%) acquired HIV within 5 years prior to enrollment in this study. The median CD4 + count was 400 cells/µl (range: 8-1977). Less than 20% of the patients had CD4 + count between 0 and 199 cells/µl. More than 90% of the patients were using HAART drugs.

**Table 1:** Socio-demographic characteristics of respondents who did have and did not have HIV related ocular disorders

Variables	HIV related ocular disorders		Total	χ^2^	*P*-value
	Yes *n* (%)	No *n* (%)	*n* (%)		
**Sex**					
Male Female	6 (5.9) 21 (5.2)	95 (94.1) 383 (94.8)	101 (100.0) 404 (100.0)	0.88	0.77
**Age**					
0-39 40+	6 (2.5) 21 (8.0)	236 (97.5) 242 (92.0)	242 (100.0) 263 (100.0)	7.55	0.006
**Religion**					
Islam & others Christianity	13 (6.4) 14 (4.6)	190 (93.6) 288 (95.4)	203 (100.0) 302 (100.0)	0.75	0.39
**Marital status**					
Never married Ever married	1 (1.6) 26 (5.9)	63 (98.4) 415 (94.1)	64 (100.0) 441 (100.0)	2.07	0.23*
**Residency**					
Rural Urban	0 (0) 27 (6.0)	57 (100.0) 421 (94.0)	57 (100.0) 448 (100.0)	3.63	0.06*
**Level of education**					
Primary and lower Secondary and above	11 (4.7) 16 (5.9)	221 (95.3) 257 (94.1)	232 (100.0) 273 (100.0)	0.31	0.58
**Occupation**					
Unemployed Employed	2 (2.9) 25 (5.7)	68 (97.1) 410 (94.3)	70 (100.0) 435 (100.0)	1.00	0.56*

* Fisher's exact test

**Table 2:** Clinical characteristics of respondents who did or did not have HIV related ocular disorders

Variables	HIV related ocular disorders		Total	χ^2^	*P*-value
	Yes *n* (%)	No *n* (%)	*n* (%)		
**Duration of HIV since diagnosis**					
<5 years >5 years	22 (5.7) 5 (4.2)	364 (94.3) 114 (95.8)	386 (100.0) 119 (100.0)	0.40	0.53
**HAART status**					
Using HAART Not using HAART	25 (5.4) 2 (5.0)	440 (94.6) 38 (95.0)	465 (100.0) 40 (100.0)	0.01	1.00*
**Duration of HAART**					
<5 years >5 years	20 (5.8) 7 (4.4)	325 (94.2) 153 (95.6)	345 (100.0) 160 (100.0)	0.44	0.51
**WHO Clinical stages**					
1&2 3&4	22 (5.1) 5 (6.8)	409 (94.9) 68 (93.2)	431 (100.0) 73 (100.0)	0.38	0.57*
**CD4+ count**					
0-199 200-499 ≥500	7 (7.4) 16 (6.8) 4 (2.3)	88 (92.6) 219 (93.2) 169 (97.7)	95 (100.0) 235 (100.0) 173 (100.0)	4.89	0.09
**Eye symptoms**					
Yes No	18 (9.0) 9 (3.0)	182 (91.0) 296 (97.0)	200 (100.0) 305 (100.0)	8.73	0.003
**Visual impairment**					
Yes No	5 (22.7) 22 (4.6)	17 (77.3) 460 (95.4)	22 (100.0) 482 (100.0)	13.69	0.004*

*Fisher's exact test

### Ocular disorders and HIV/AIDS

A total of 200 (39.6%) participants had at least one eye-related complaint during assessment. The complaints were mainly related to blurred vision (23.2%) and pain (4.6%). However, 272 (53.9%; 95% C.I.= 49.6%-58.2%) participants had at least one ocular disorder in either or both eyes after ocular examination. HIV-related ocular disorders were found among 27 patients, giving a prevalence of 5.4%.

**Fig. 1:**
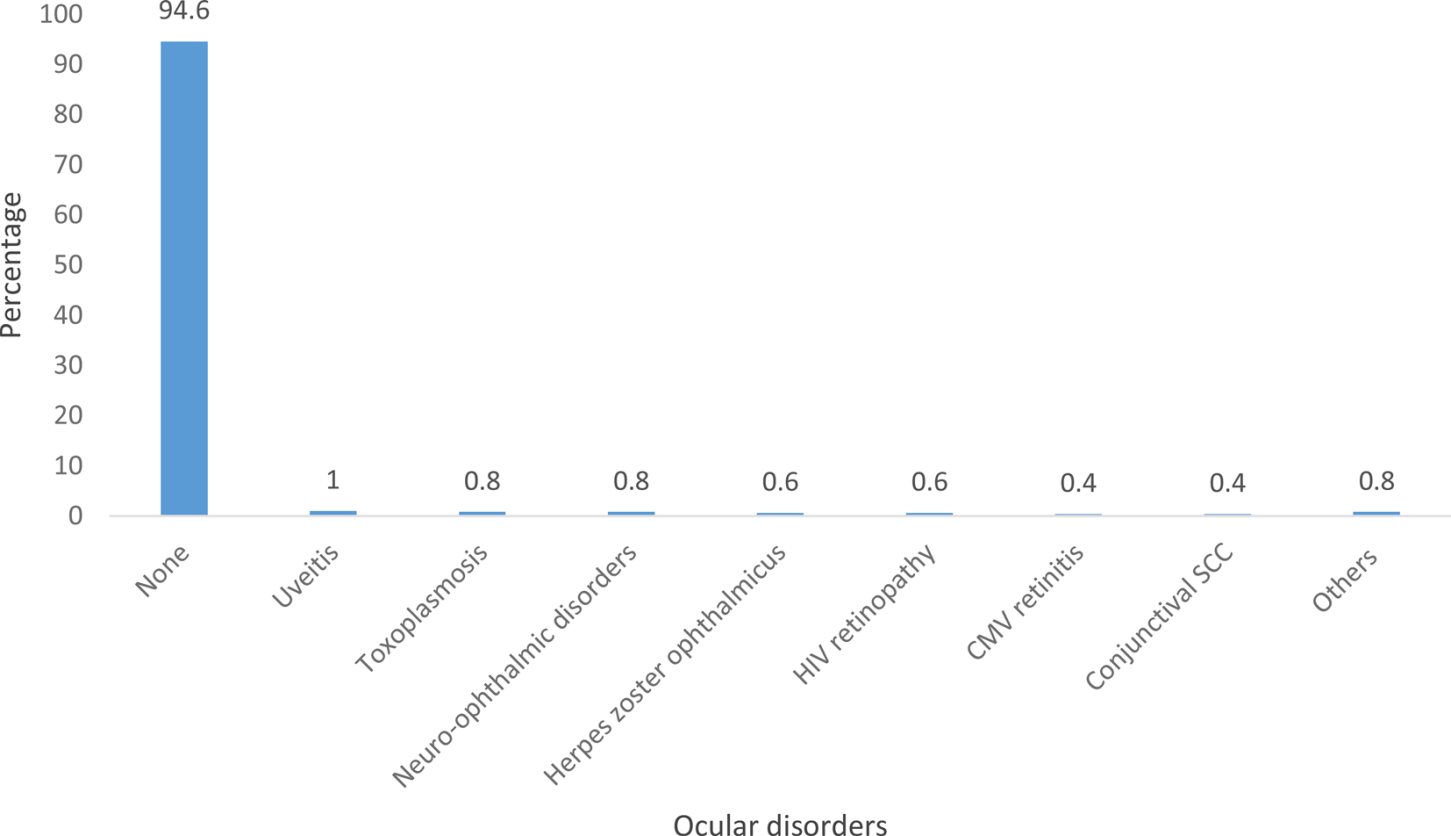
HIV related ocular disorders among respondents. SCC = Squamous cell carcinoma

**Fig. 2:**
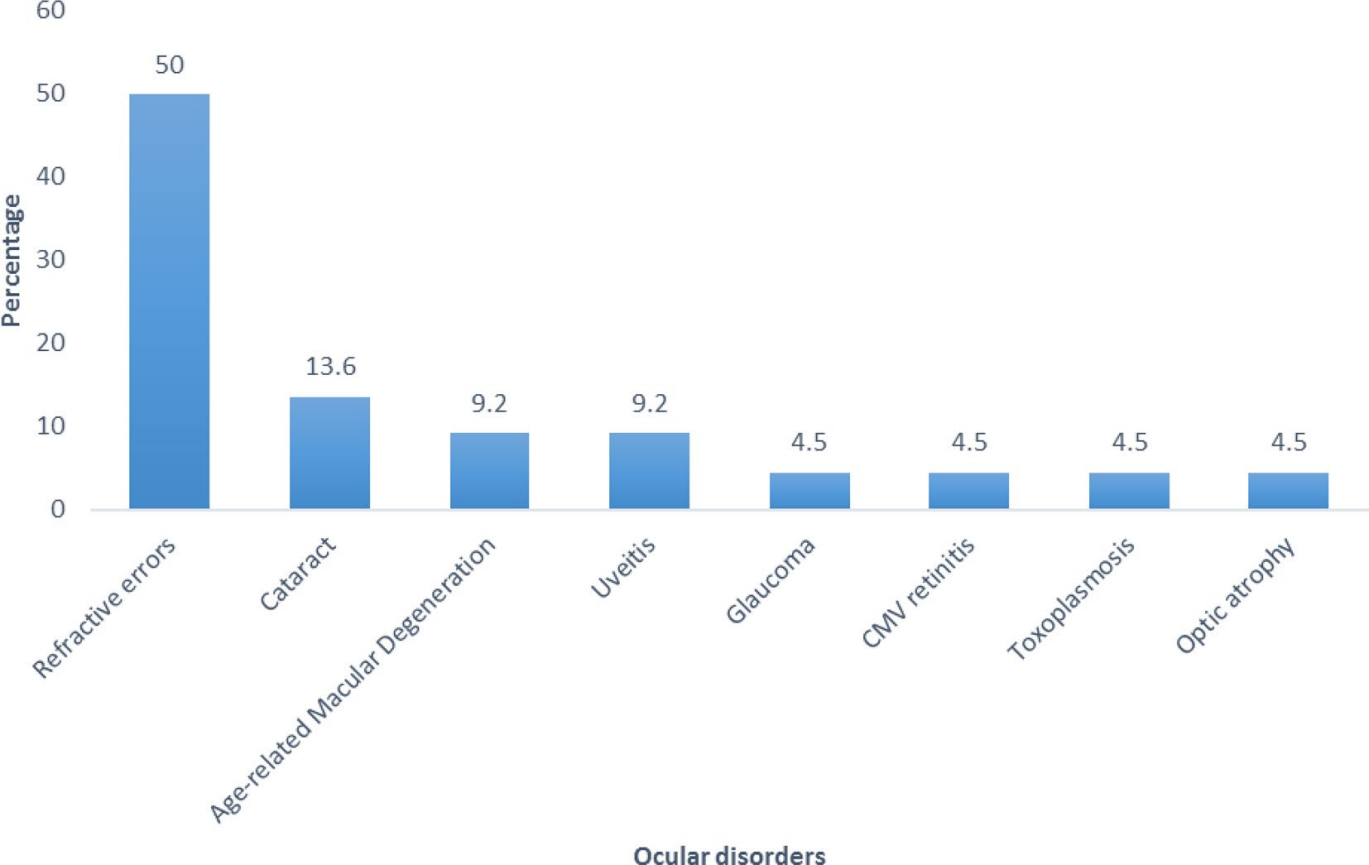
Causes of visual impairment among respondents

**Fig. 3:**
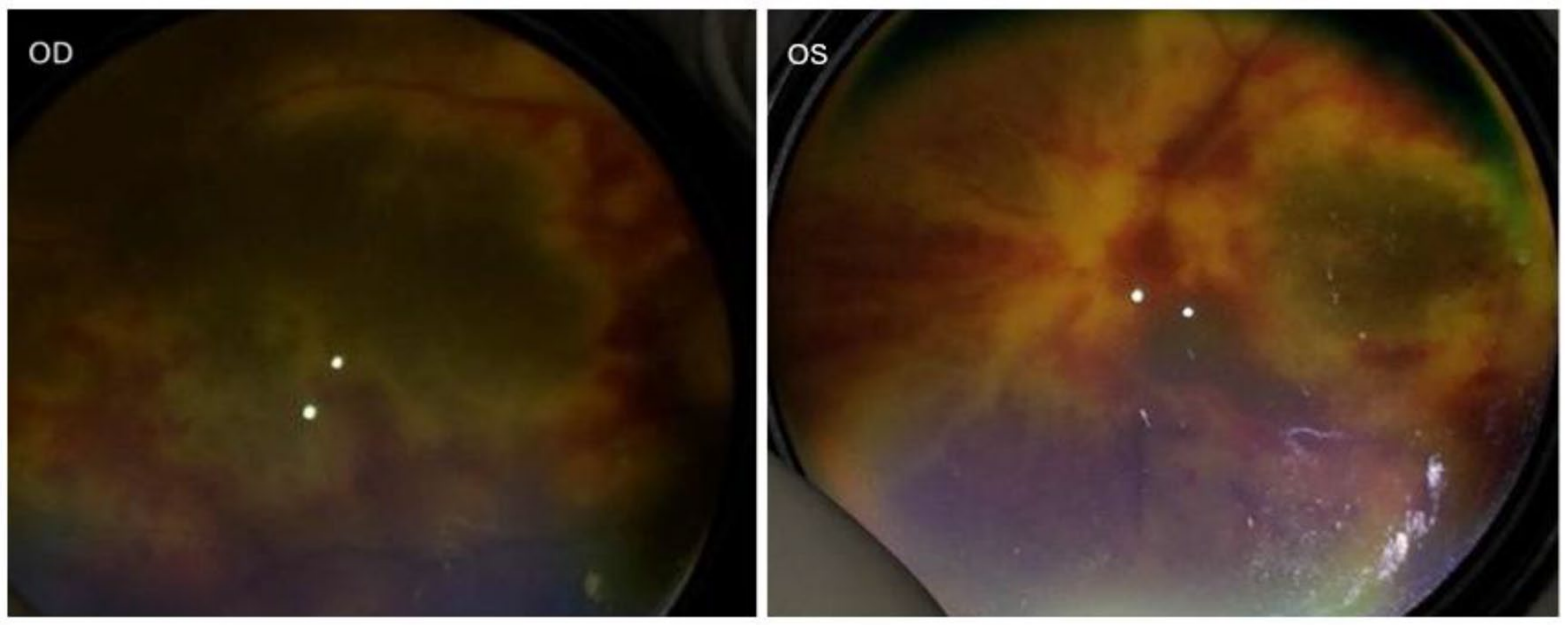
Colour fundus photograph of both eyes of a participant with severe cytomegalovirus retinitis

The commonest HIV related disorders were uveitis (1%), presumed toxoplasmosis (0.8%) and neuro-ophthalmic disorders (0.8%). Other HIV-related disorders included herpes zoster ophthalmicus (HZO) (0.6%), HIV retinopathy (0.6%), cytomegalovirus (CMV) retinitis (0.4%) and presumed conjunctival squamous cell carcinoma (SCC) (0.4%) (Fig. 1). Six participants with HIV-related ocular disorders had tuberculosis. Some participants (48.5%) had ocular disorders that were not related to HIV/AIDS. Conjunctival degenerative disorders (pterygium and pingueculum) constituted the commonest non-HIV related disorders found among these participants. Other non-HIV related disorders such as refractive errors, glaucoma and cataract were also found among the participants. Most (95.6%) participants had normal vision in their better eyes while 22 (4.4%; 95% C.I.=2.6%-6.2%)) were visually impaired. Eighteen participants had moderate visual impairment while 4 were blind. The main causes of visual impairment among the participants were refractive errors and cataract (Fig. 2). It is noteworthy that one of the two participants with CMV retinitis (Fig. 3) eventually lost his vision despite intravitreal injection of ganciclovir because of delayed diagnosis.

### Predictors of HIV related ocular disorders and visual impairment among respondents

Patients with CD4 + count of at least 500 cells/µl were about four times less likely to develop HIV related ocular disorders than those with CD4 + count < 200 cells/µl (OR = 0.24; 95% C.I.=0.07–0.89; *p* = 0.03). Those without visual impairment were about four times less likely to have HIV-related ocular disorders than those with visual impairment (OR = 0.25; 95% C.I. = 0.08–0.80; *p* = 0.02). Further, those who did not have eye complaints were about three times less likely to develop HIV related ocular disorders than those who had eye complaints (OR = 0.35, 95% C.I.=0.15–0.83; *p* = 0.02) (Tables 3 and 5).

**Table 3:** Visual impairment and socio-demographic characteristics of respondents

Variables	Visual impairment		Total	χ^2^	*P*-value
	Yes *n*(%)	No *n*(%)	*n*(%)		
**Sex**					
Male Female	7 (6.9) 15 (3.7)	94 (93.1) 388 (96.3)	101 (100.0) 404 (100.0)	2.00	0.17*
**Age**					
0-39 40+	2 (0.8) 21 (7.6)	239 (99.2) 243 (92.4)	241 (100.0) 263 (100.0)	13.83	0.0001
**Religion**					
Islam & others Christianity	8 (4.0) 14 (4.6)	194 (96.0) 288 (95.4)	202 (100.0) 302 (100.0)	0.13	0.72
**Marital status**					
Never married Ever married	3 (4.8) 19 (4.3)	60 (95.2) 422 (95.7)	64 (100.0) 441 (100.0)	0.03	0.75*
**Residency**					
Rural Urban	3 (5.3) 19 (4.3)	54 (94.7) 428 (95.7)	57 (100.0) 447 (100.0)	0.12	0.73*
**Level of education**					
Primary and lower Secondary and above	14 (6.1) 8 (2.9)	217 (93.9) 265 (97.1)	231 (100.0) 273 (100.0)	2.94	0.09
**Occupation**					
Unemployed Employed	6 (8.7) 16 (3.7)	63 (91.3) 419 (96.3)	69 (100.0) 435 (100.0)	3.59	0.10*

* Fisher's exact test

**Table 4:** Logistic regression analysis for HIV related ocular disorders

Variable		Odds Ratio	95% C.I.
CD4+ count	0-199 (ref) 200-499 ≥500	0.75 0.24*	0.29-1.95 0.07-0.89
Eye complaints	Yes (ref) No	0.35*	0.15-0.83
Visual impairment	Yes (ref) No	0.25*	0.08-0.80
Age-groups	0-39 (ref) 40+	2.32	0.88-6.11

*p<0.05

Presence of both HIV-related and non-HIV related ocular disorders and advanced stage of HIV/AIDS disease were independent predictors of visual impairment in this study (Tables 4 and 6). Respondents without HIV-related ocular disorders were about 20 times less likely to have visual impairment compared to those with HIV-related ocular disorders (OR = 0.05, 95% C.I.=0.01–0.28; *p* = 0.001). Participants who did not have non-HIV related ocular disorders were about eight times less likely to have visual impairment compared to those who had non-HIV related ocular disorders (OR = 0.13, 95% C.I.=0.03–0.63; *p* = 0.01). Those who belonged to WHO clinical stages 1&2 were about three times less likely to have visual impairment than participants in the WHO clinical stages 3&4 HIV disease (OR = 0.35, 95% C.I.=0.13–0.95; *p* = 0.04).

**Table 5:** Visual impairment and clinical characteristics of respondents

Variables	HIV related ocular disorders		Total	χ^2^	*P*-value
	Yes *n*(%)	No *n*(%)	*n*(%)		
**Duration of HIV since** **diagnosis**					
<5 years >5 years	13 (3.4) 9 (7.6)	372 (96.6) 110 (92.4)	385 (100.0) 119 (100.0)	3.82	0.05
**HAART status**					
Using HAART Not using HAART	21 (4.5) 1 (2.6)	444 (95.5) 38 (95.0)	465 (100.0) 39 (100.0)	0.33	1.00*
**Duration of HAART**					
<5 years >5 years	11 (3.2) 11 (6.9)	334 (96.8) 148 (93.1)	345 (100.0) 159 (100.0)	3.63	0.06
**WHO Clinical stages**					
1&2 3&4	15 (3.5) 7 (9.6)	415 (96.5) 66 (90.4)	431 (100.0) 73 (100.0)	5.55	0.03*
**CD4+ count**					
0-199 ≥200	5 (5.3) 17 (4.1)	89 (94.7) 393 (95.9)	94 (100.0) 410 (100.0)	0.25	0.62*
**Eye symptoms**					
Yes No	14 (7.0) 8 (2.6)	186 (93.0) 296 (97.4)	200 (100.0) 304 (100.0)	5.52	0.02
**Non-HIV ocular disorders**					
Yes	17 (6.9)	230 (93.1)	247 (100.0)	7.35	0.01
No	5 (1.9)	252 (98.1)	257 (100.0)		
**HIV ocular disorders**					
Yes No	5 (18.5) 17 (3.6)	22 (81.5) 460 (96.4)	27 (100.0) 477 (100.0)	13.69	0.004*

*Fisher's exact test

**Table 6:** Logistic regression analysis for visual impairments among PLWHA

Variable		Odds Ratio	95% C.I.
Eye complaints	Yes (ref) No	0.54	0.21-1.42
HIV-related ocular disorders	Yes (ref) No	0.05*	0.01-0.28
Non HIV-related ocular disorders	Yes (ref) No	0.13*	0.03-0.63
Education	Primary & below (ref) Secondary & above	0.59	0.23-1.54
Duration of HAART	≥5 years (ref) <5 years	0.59	0.20-1.76
Duration of HIV	≥5 years (ref) <5 years	0.70	0.23-2.17
WHO clinical stage	3&4 (ref) 1&2	0.35*	0.13-0.95
Age-groups	0-39 (ref) ≥40	4.26	0.93-19.57

*p<0.05

## Discussions

This is the first multicentre study of ocular problems among persons living with HIV/AIDS in a subnational geographic area of Nigeria. The overall prevalence of ocular disorders in this study was lower than the prevalence figures reported from similar studies in Calabar [11] and Lagos [18], cities in Nigeria where 72.0% and 78.5% of the PLWHA had ocular disorders in one or both eyes. All participants with HIV-related ocular disorders were not suffering from any known cause of immunosuppression at the time of the study except six who had tuberculosis, the single most important HIV-related opportunistic infection in developing countries such as Nigeria [26, 27].

The prevalence of HIV-related ocular disorders found in this study is lower than that reported in most similar studies conducted around the world, except in the Cape Coast, Ghana where about 6% of the study participants had HIV-related pathologies [12]. Prevalence of HIV-related ocular disorder was as high as 23.8% in the US at the time of enrollment of participants into the multicentre Study on Ocular Complications of AIDS (SOCA) [6]. The prevalence in the US dropped significantly after the commencement of HAART as exemplified by reduction in the rate of new-onset CMV retinitis [16, 28]. In India, prevalence of HIV-related ocular disorders varied between 33.69% in Bhubaneswar, Eastern India [13, 29] to 43.0% in Amritsar, Northwestern India [29]. In Nigeria, prevalence of HIV-related ocular disorders varied between 7.1% in Benin-City [10], South-South Nigeria to 14.3% in Owo [25], South-West Nigeria. Recent studies in Ibadan [30] and Calabar [11] estimated the prevalence of HIV-related disorders at 10% and 30.9% respectively. However, lower prevalence (2.2%) was reported from a study in Northern Romania [31]. The low prevalence of HIV-related ocular disorders in this study was probably because most (85.5%) participants had early WHO clinical stages (1&2) of HIV infection. In addition, 92.1% of them were on HAART which they had used for a median period of 2.2 years at the time of this study. The major HIV-related ocular disorders found in this study were similar but with lower prevalence compared to those reported from other states of Nigeria [10, 11, 30, 32] and other African countries [12, 33, 34]. Opportunistic infections such as HZO, toxoplasmosis were the commonest HIV-related ocular disorders in most parts of Nigeria. Cytomegalovirus retinitis had been reported to be rare in most African countries [35]. Contrary to this notion, the prevalence of CMV retinitis has been on the increase with the widespread availability of HAART. The prevalence of CMV retinitis was respectively 0.4%, 0.7%, 1.6%, and 1.8% in present study (Osun), Yenagoa [36], Ibadan [32] and Lagos [18]. The rising prevalence of CMV retinitis in Nigeria has grave implication for vision among the PLWHA. Anti-CMV drugs are difficult to find in Africa, and there is increased risk for the development of ocular IRIS among this patient when HAART are initiated.

Visual impairment was less common in this study (4.4%) than in Benin-City (23.3%) [19], Ibadan (19.6%) [30], Yenagoa (10.8%) [36], and Lagos (8.6%) [18]. Although causes of visual impairments, which included refractive errors, cataract, and glaucoma, in all the studies, variations in prevalence might result from different age distributions. Visual impairment could compound the psychosocial problems faced by PLWHA. Yermukhanova had suggested that some PLWHA may not seek treatment at Eye Clinics for ocular conditions due to perceived stigma related to their HIV status [37].

Correlates of HIV-related ocular disorders in this study were consistent with findings of the study in Felege, North Western Ethiopia [22]. In Ethiopia, patients who had eye complaints were about 11 times more likely to have HIV related ocular disorders; those with CD4 + count < 200 cells/µl were about seven times more likely to have ocular complications of HIV, and patients with visual impairment at presentation were about four times more likely to develop HIV related ocular disorders than those who did not. Although Pathai et al. [38] had earlier reported that PLWHA with WHO clinical stages 3&4 disease were nine times more likely to develop HIV related ocular disorders, such association was not found in this study. Rather, WHO clinical stages of HIV disease were associated with visual impairment. Presence of any ocular disorder and advanced HIV/AIDS disease predispose patients to visual impairment. However, neither viral load nor CD4 + count was statistically associated with presence of HIV related ocular disorder in Ibadan study [30]. That this study was conducted among at risk subjects in a clinical setting is a major limitation. A population-based study would have given a more accurate description of the epidemiology of ocular disorders among PLWHA.

## Conclusion

Significant proportion of HIV/AIDS patients in Osun State, Nigeria had ocular disorders. Some of these disorders resulted in visual impairment which could impact negatively on the quality of life of the patients. Occurrence of ocular disorder was associated with older age, CD4 + count less than 200 cells/µL, presence of eye complaints and visual impairment. On the other hand, occurrence of any ocular disorder and advanced clinical stage of HIV/AIDS (and its attendant severe immune suppression) were likely to predispose patients to development of visual impairment.

It is therefore very important that due attention is paid to the eye health of PLWHA with advanced stages of HIV/AIDS and severe immune suppression with the aim of ensuring early diagnosis and treatment of eye diseases before visual impairment develops. Thus, genuine attempt at improving the quality of life of PLWHA should include provision of barrier-free eye care services to maximise the gains of HAART drugs. Further research is necessary to unravel the cause of geographic variations in HIV-related ocular disorders which have persisted even with the availability of HAART in developing countries.

## Supplementary Information

Below is the link to the electronic supplementary material.

Supplementary Material 1

## Data Availability

The data and materials that support the findings of this research are not publicly available but can be obtained from the corresponding author (O.U.K).

## Abbreviations

AIDS: Acquired Immune Deficiency Syndrome
ART: Anti-retroviral Treatment
CD: Cluster of Differentiation
CMV: Cytomegalovirus
D: Dioptre
HAART: Highly Active Anti-Retroviral Therapy
HIV: Human Immunodeficiency Virus
HZO: Herpes Zoster Ophthalmicus
IRIS: Immune Reconstitution Inflammatory Syndrome
LAUTECH: Ladoke Akintola University of Technology
OR: Odd Ratio
PL: Perception of Light
PLWHA: Person Living with HIV/AIDS
SCC: Squamous Cell Carcinoma
VA: Visual Acuity
WHO: World Health Organisation
