# Supplementary material for: Ocular disorders among persons living with HIV/AIDS in Osun State, Nigeria: a cross-sectional study
Source: BMC Ophthalmol. 2026 Mar 11;26:127. doi: 10.1186/s12886-026-04689-w (PMC13352754; doi:10.1186/s12886-026-04689-w)
Supplement: Supplementary file 1 — Supplementary Material 1 [file 12886_2026_4689_MOESM1_ESM.docx]

**QUESTIONNAIRE**

**OCULAR DISORDERS AMONG PLWHA IN OSUN STATE, NIGERIA**

**Serial No: Hospital No: ARV Treatment centre:**

**GSM NO:**

**Date of interview and examination:**

1. **SOCIODEMOGRAPHIC CHARACTERISTICS**
2. Age (as at last birthday): _ _ _ years
3. Sex: 1. Male 2. Female
4. Religion: 1. Christianity 2. Islam 3. Traditional religion 4. Others (specify)…………………..
5. Ethnicity: 1. Yoruba 2. Igbo 3. Hausa 4. Fulani 5. Others (specify)……………………..
6. Marital status: 1. Single 2. Married 3. Divorced 4. Separated 5. Widowed 6. Living with partner 7. Partner deceased
7. Family set up: 1. Monogamous 2. Polygamous 3. No formal marriage
8. Place of domicile: ……………………………………………..
9. Highest education level attained: 1. No formal education 2. Primary school 3. Secondary 4. Tertiary 5. Quranic only 6. Others (specify) ………………….…….
10. Employment status: 1. Unemployed 2. Housewife 3. Retired 4. Student 5. Farmer

6. Artisan 7. Trader 8. Driver 9. Professional 10. Others (specify)…………………..

1. **HIV/AIDS-RELATED CHARACTERISTICS**
2. Which month and year did you first test positive for HIV/AIDS? _ _ /_ _
3. How old were you when the diagnosis of HIV/AIDS was made? _ __ _ years
4. How did you contract HIV infection? 1. Heterosexual contact 2. Homosexual contact 3. Blood transfusion 4. Sharing injection needles 5. Sharing other sharp objects 6.Others (specify) ………………………
5. Which diseases have you suffered since you tested positive for HIV?..............................................
6. Have you suffered from any of the following diseases since you tested positive for HIV? 1. Tuberculosis 2. Syphilis 3. Pneumonia 4. Hypertension 5. Diabetes 6. Gonorrhoea 7. Others (specify)………………………
7. Are you currently receiving treatment for any of the following diseases?

1. Tuberculosis 2. Syphilis 3. Pneumonia 4. Hypertension 5. Diabetes 6. Gonorrhoea 7. Others (specify)………………………

1. Are you receiving any treatment/ drug now for HIV/AIDS? 1. Yes 2. No
2. If yes, when did you start the treatment? ----/----
3. How often do you use your HIV drugs? 1. Daily 2. Every other day 3. Once a week 4. Anytime
4. Indicate reason(s) why you could not use your HIV drugs at times:

1. Always out of stock 2. Hospital/pharmacy too far 3. Hospital strike 4. Adverse drug reaction 5. Nobody to help 6. Others (specify)…………………………….

1. **EYE PROBLEMS IN HIV/AIDS**
2. Have you had any eye complaints since tested positive for HIV? 1. Yes 2. No
3. If yes, which of the following eye complaints do you have? 1. Reduction in vision 2. Ocular pains 3. Floaters 4. Ulcer/skin eruptions around the eye 5. Others (specify)……………………
4. Are you receiving treatment for any eye problem now? 1. Yes 2. No
5. Where are you receiving treatment for eye problem now? 1. LAUTECH 2. OAUTHC

3. Nowhere 4. Pharmacy/Chemist 5. Others (specify): ………………………………………..

1. **INFORMATION FROM PATIENT RECORDS**
2. Mode of HIV transmission of HIV to the patient?

1. Heterosexual contact

2. Homosexual contact

3. Blood transfusion

4. Intravenous drug use

5. Others (specify) …………………………….

1. WHO clinical staging? …………………………………….
2. HAART drugs patient is receiving from records

1.

2.

3.

1. Other drugs patient is receiving currently

1.

2.

3.

1. Recent/Current CD+ T- lymphocyte count: ………………/µL as at Date: ………………………
2. Viral load…………………………………../µL as at Date: ………………………………….
3. HIV Strain……………………………
4. Date HIV/AIDS detected/diagnosed? ……………………….
5. Co-morbid systemic or sexually transmitted disorders

1. 2.

3. 4.

1. **OCULAR EXAMINATIONS OD OS**
2. **Distance VA**
3. Unaided
4. With pinhole
5. With glasses
6. **Ocular alignment**
7. Straight eyes
8. Exotropia
9. Esotropia
10. Others (specify) ……………………… ……………………………
11. **Ocular motility**
12. Full
13. Some restriction
14. No movement at all
15. **Lids and Lashes**
16. Normal
17. Ptosis
18. Oedema
19. Discharge
20. Vesicles
21. Papular rashes
22. Lesions (State name)
23. Others (specify) ………………………….. …………………………………
24. **Conjunctivae**
25. Normal (White)
26. Injection
27. Lesion (state name)
28. Others (specify) ……………………………. ……………………………………
29. **Sclerae**
30. Normal
31. Lesions (specify)
32. **Corneae**  **OD OS**
33. Clear
34. Ulcer
35. Oedema
36. Opacity
37. Others (specify) ……………………………….. …………………………
38. **Anterior chamber**
39. Deep and Quiet
40. Shallow
41. Hypopyon
42. cells
43. **Iris**
44. Atrophy
45. Neovascularization
46. Nodules
47. Others (specify) ………………………………… ………………………………..
48. **Pupils**
49. Round and Reactive
50. RAPD
51. TAPD
52. Sluggish
53. Others (specify) ………………………………… …………………………………….
54. **Lens**
55. Clear
56. Cataract
57. Subluxated
58. Dislocated
59. No lens
60. Intraocular lens
61. Others (specify) …………………………….. ……………………………………..
62. **Intraocular pressures (mmHg)**
63. **Vitreous**  **OD OS**
64. Cells
65. Strands/bands
66. Haemorrhage
67. Exudates (as in endophthalmitis)
68. Others (specify) …………………………………… …………………………...
69. **Retina**
70. Normal
71. Cotton wool spots
72. Haemorrhages
73. Exudates
74. Retinitis
75. Choroiditis
76. Retinal detachment
77. Chorioretinal scars
78. Others (specify) ………………………………… ………………………….
79. **Macula**
80. Normal
81. Oedema
82. Haemorrhage
83. Others (specify) ………………………………….
84. **Optic Disc**
85. Normal
86. C/D ratio
87. Oedema
88. Pallor
89. Haemorrhage
90. Others (specify) ………………………………… ………………………………
91. **DIAGNOSES**
92. **Final HIV-defining Diagnosis OD OS**
93. Herpes Zoster Ophthalmicus
94. Squamous Cell Carcinoma
95. Blepharitis
96. Herpes simplex keratitis
97. Immune Recovery Uveitis
98. Kaposis’ Sarcoma
99. HIV Retinopathy
100. CMV Retinitis
101. B-cell Lymphoma
102. Toxoplasmal retinochoroiditis
103. Others (specify) ………………………………………. ………………………….
104. **General ocular diagnosis**
105. Cataract
106. Glaucoma
107. Refractive errors
108. Age-related macular degeneration
109. Others (specify) …………………………………………. …………………………..
110. **RECOMMENDATIONS/ADVICE**
